# An LLM-supported just-in-time adaptive intervention delivered by a socially assistive robot for cognitive training in mild cognitive impairment: study protocol for an assessor-blinded randomized controlled trial

**DOI:** 10.3389/fdgth.2026.1897477

**Published:** 2026-08-28

**Authors:** Jingzheng Yan, Yahui Cheng, Yaning Wang, Yingjuan Cao

**Affiliations:** 1 School of Nursing and Rehabilitation, Shandong University, Jinan, China; 2 Department of Nursing, Qilu Hospital, Shandong University, Jinan, China; 3 Nursing Theory & Practice Innovation Research Center, Shandong University, Jinan, China

**Keywords:** cognitive training, digital therapeutics, just-in-time adaptive intervention, large language model, mild cognitive impairment, neuroimaging biomarkers, randomized controlled trial, socially assistive robot

## Abstract

**Background:**

Mild cognitive impairment (MCI) is a clinically important stage at which non-pharmacological interventions may help preserve cognitive function and daily functioning. Existing digital cognitive training programs are often limited by static task progression, declining engagement, and insufficient responsiveness to moment-to-moment cognitive and emotional states.

**Objective:**

This study protocol describes a single-center, parallel-group, assessor-blinded randomized controlled trial evaluating an adaptive cognitive training intervention delivered by a socially assistive robot and supported by a large language model (LLM).

**Methods and analysis:**

A total of 126 adults aged 60 years or older with MCI will be randomized 1:1 to either an LLM-supported robot-assisted adaptive cognitive training intervention (*n* = 63) or a matched non-adaptive computerized cognitive training control condition (*n* = 63). Both groups will receive three individually delivered sessions per week for 24 weeks, with each session lasting approximately 45–60 min, followed by a 12-week post-intervention follow-up to assess durability. The intervention group will receive multi-domain cognitive training through a socially assistive robot, with the LLM used as a bounded communication module that generates supportive language for controller-selected task adaptation, instruction style, feedback, and motivational support according to prespecified performance and affective indicators. The primary outcome is change in Montreal Cognitive Assessment (MoCA) score from baseline to week 24. Maintenance of cognitive change at week 36 will be analyzed as a secondary exploratory durability outcome. Secondary outcomes include domain-specific cognitive performance, emotional symptoms, motivation, engagement, adherence, usability, acceptance, expectancy and novelty effects, and safety. A nested exploratory imaging-biomarker sub-cohort will undergo resting-state functional magnetic resonance imaging and plasma biomarker assessment to explore changes in functional connectivity and neurobiological markers. Analyses will follow the intention-to-treat principle using mixed-effects models for repeated measures.

**Ethics and dissemination:**

The protocol has been approved by the Ethical Committee of the School of Nursing and Rehabilitation, Shandong University (Approval No. 2026-R-072; approved March 10, 2026). Written informed consent will be obtained before enrollment.

**Clinical Trial Registration:**

https://www.chictr.org.cn/historyversionpubEN.html?regno=ChiCTR2600125870, identifier ChiCTR2600125870.

## Introduction

Mild cognitive impairment (MCI) is a clinically meaningful intermediate state between expected cognitive aging and dementia. It is associated with measurable cognitive decline, elevated risk of functional deterioration, and increased likelihood of progression to Alzheimer disease or other dementias (1, 2). Because pharmacological options for MCI remain limited and disease-modifying treatment pathways are still evolving, non-pharmacological strategies such as cognitive training and cognitive stimulation remain central to early intervention research (3–5).

Computerized cognitive training can be scalable, repeatable, and relatively low risk, but clinical effects have been inconsistent. Several barriers are particularly relevant for older adults with MCI: static task sequences, limited personalization, low ecological validity, fatigue, and progressive decline in engagement during repeated training (4, 6, 7). These limitations suggest that digital cognitive interventions should not only deliver cognitive challenge but also respond to the user state during the session.

Socially assistive robots may improve the human-centered qualities of digital interventions by providing embodied interaction through speech, gaze, gesture, and affective cues. Prior work in geriatric care and rehabilitation suggests that robot-assisted systems can be acceptable to older adults and may support engagement, social presence, and motivation (8–10). However, many existing robot systems still rely on scripted or rule-based dialogue, which restricts their ability to support flexible conversation, sustained personalization, and affect-sensitive interaction across repeated sessions.

Recent progress in large language models (LLMs) provides a technical pathway for more context-aware and adaptive interaction in digital health interventions (11, 12). In parallel, the just-in-time adaptive intervention (JITAI) framework offers a behavioral science model for delivering support when and how it is needed, based on dynamically changing individual states (13). For clinical trials involving artificial intelligence, the SPIRIT-AI extension emphasizes transparent reporting of the AI intervention, input and output data, human-AI interaction, error handling, and version control (14). These reporting principles are especially relevant when an LLM is embedded in a health-related intervention for cognitively vulnerable participants.

This protocol describes a randomized controlled trial of an LLM-supported socially assistive robot for adaptive cognitive training in older adults with MCI. The intervention combines a cognitive adaptation loop, which adjusts task difficulty based on real-time performance, with an affective-motivational adaptation loop, which adjusts prompting and emotional support based on observed or inferred user state. The study aims to evaluate the clinical effect of the adaptive intervention compared with a matched non-adaptive active control condition and to explore potential neurobiological correlates through a nested imaging and fluid biomarker sub-cohort.

Beyond global cognition, older adults with MCI may also experience depressive symptoms, anxiety, apathy, and reduced initiative, which can influence adherence, motivation, and responsiveness to cognitive interventions (15–19). These symptoms are therefore relevant both as secondary outcomes and as user-state indicators for adaptive intervention delivery.

Evidence from lifestyle, cognitive training, and ecologically oriented rehabilitation research suggests that active, socially integrated, and multi-domain approaches may support cognitive performance and everyday functioning, although transfer effects and neural mechanisms remain incompletely understood (20–28). These considerations support the use of a multi-domain training program with engagement, functional relevance, and exploratory neurobiological outcomes built into the protocol.

## Methods and analysis

### Study design and setting

This is a prospective, single-center, parallel-group, assessor-blinded randomized controlled trial with a 1:1 allocation ratio. A total of 126 participants will be randomized, with 63 participants assigned to each group. Participants will be assigned to either an LLM-supported robot-assisted adaptive cognitive training group (AI-Robot group) or an active control group receiving non-adaptive computerized cognitive training. The intervention period will last 24 weeks, with assessments at baseline, immediately after completion of the intervention (week 24), and 12 weeks after intervention completion (week 36). A nested exploratory imaging-biomarker sub-cohort will undergo resting-state functional magnetic resonance imaging (rs-fMRI) and plasma biomarker testing at baseline and week 24.

**Figure 1:**
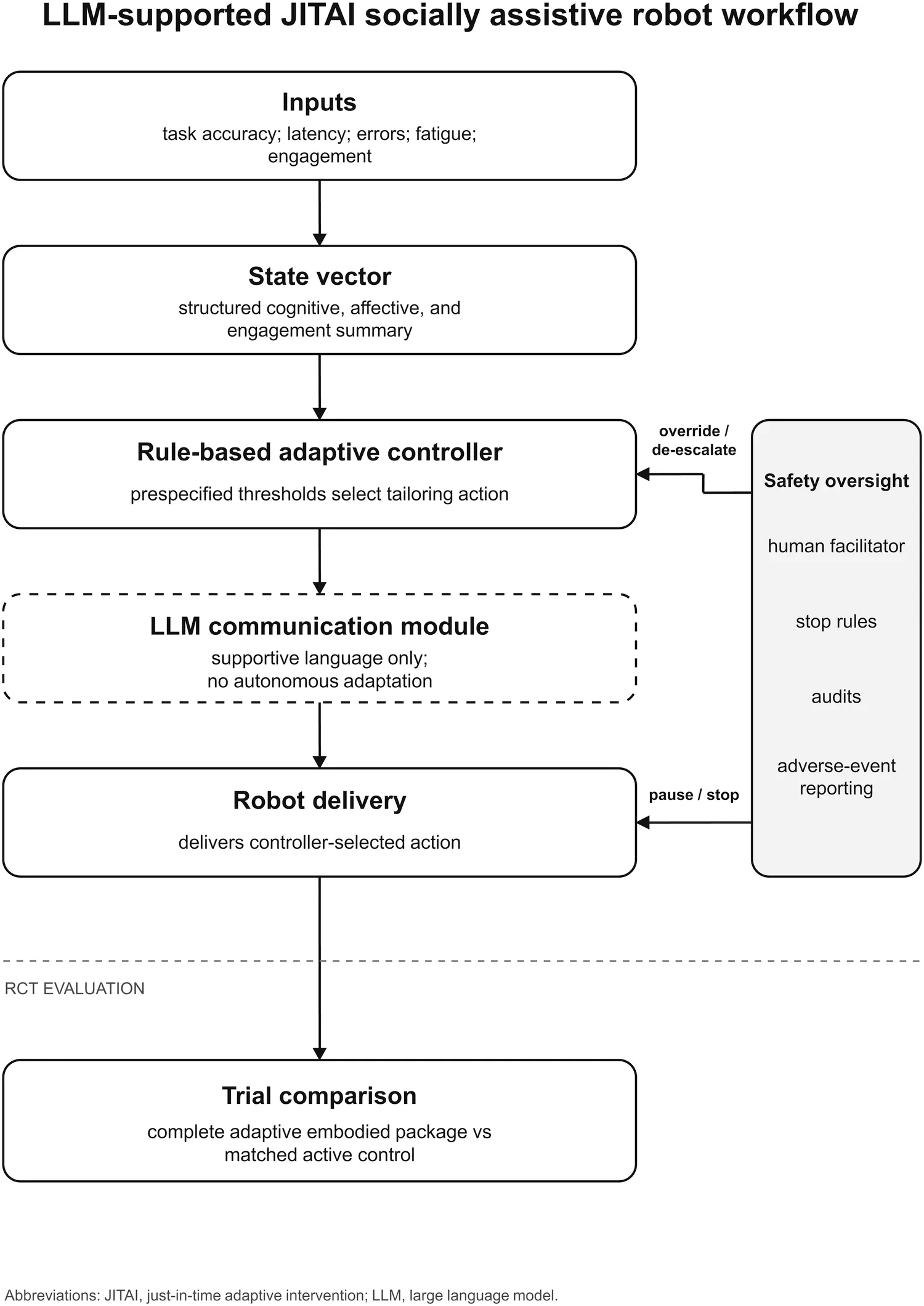
Conceptual workflow of the LLM-supported JITAI socially assistive robot cognitive training intervention. Task performance and behavioral-affective inputs are summarized into a structured user-state vector. A prespecified rule-based adaptive controller selects the tailoring action. The LLM communication module generates bounded supportive language only and cannot autonomously alter the selected action. The socially assistive robot delivers the controller-selected intervention. Parallel human safety oversight can override or de-escalate the controller and pause or stop robot delivery. The complete adaptive embodied package will be compared with a matched active control.

The protocol has been structured to align with SPIRIT 2013 and the SPIRIT-AI extension for trial protocols involving artificial intelligence components (14, 29). The trial has been registered with the Chinese Clinical Trial Registry (ChiCTR; ChiCTR2600125870; prospective registration; https://www.chictr.org.cn/historyversionpubEN.html?regno=ChiCTR2600125870). The trial will be conducted at the Community Health Service Center of Baotuquan Subdistrict, Lixia District, Jinan, Shandong Province, China, and associated community-based research settings. Protocol version: Version 1.0, March 10, 2026. Study status at resubmission (July 2026): not yet recruiting. The conceptual workflow of the intervention is shown in Figure 1.

### Trial status

At the time of resubmission (July 2026), the trial had been prospectively registered but recruitment had not yet started. The planned overall study period is March 10, 2026, to June 30, 2028. Recruitment is expected to begin after resubmission and completion of the relevant approval procedures; the anticipated recruitment period has therefore been revised to September 1, 2026, to September 30, 2027, and the trial registry will be updated accordingly before enrollment begins. Each participant will be followed for 252 days, covering the 24-week intervention period, the week 24 post-intervention assessment, and the 12-week post-intervention durability follow-up at week 36.

### Objectives and hypotheses

The primary objective is to evaluate whether 24 weeks of LLM-supported robot-assisted adaptive cognitive training improves global cognitive function, measured by the MoCA, compared with a matched non-adaptive computerized cognitive training control condition.

Secondary objectives are to examine effects on domain-specific cognitive performance, emotional symptoms, motivation and engagement, intervention adherence, usability, acceptance, safety, and durability of cognitive effects at 12-week post-intervention follow-up. The exploratory mechanistic assessment will examine whether changes in cognitive outcomes are accompanied by changes in rs-fMRI connectivity involving the default mode network (DMN) and executive control network (CEN), and by changes in plasma biomarkers related to synaptic plasticity and neurodegeneration.

The main hypothesis is that participants in the AI-Robot group will show greater improvement in MoCA scores from baseline to week 24 than participants in the active control group. Exploratory hypotheses are that the AI-Robot group will demonstrate higher engagement, better adherence, fewer overload-related session interruptions, better maintenance of cognitive gains at week 36, and favorable changes in neural connectivity and biomarker profiles.

### Participants and recruitment

Participants will be older adults with clinically diagnosed MCI. Recruitment will be conducted at the Community Health Service Center of Baotuquan Subdistrict, Lixia District, Jinan, Shandong Province, China, and through affiliated community health and local aging-service networks. Recruitment has not yet started and is expected to begin after resubmission and completion of the relevant approval procedures. The anticipated recruitment period is September 1, 2026, to September 30, 2027. Potential participants will receive oral and written information about the study, and trained research staff will assess eligibility before obtaining written informed consent. Participants may withdraw at any time without affecting access to usual care or community services.

For participants with borderline decisional capacity, consent procedures will follow local ethical requirements. A family member or legally authorized representative may be involved when required by institutional policy, but participants will be included only when they can express willingness to take part in the study procedures.

### Eligibility criteria

Inclusion criteria are as follows:

- Age 60 years or older.
- Meeting Petersen's diagnostic criteria for mild cognitive impairment based on clinical evaluation.
- MoCA score between 18 and 25 as a study-specific cognitive screening criterion after applying the original MoCA education correction (+1 point for participants with 12 years of education or fewer), with MCI confirmed clinically according to Petersen's criteria (1, 2, 30).
- Having sufficient language communication, hearing, and vision abilities to understand study instructions and complete assessments and training, with aids if needed.
- Living in a community or home setting and able to attend the 24-week cognitive training program as scheduled.
- Able and willing to provide written informed consent. Willingness and eligibility for MRI will be required only for the nested imaging-biomarker sub-cohort; refusal of MRI or MRI contraindication will not affect eligibility for the main randomized trial.

The MoCA range of 18–25 is a study-specific eligibility threshold intended to enrich the sample for mild objective cognitive impairment while reducing inclusion of participants with probable normal cognition at the upper boundary or dementia-level impairment at the lower boundary; it is not used as a standalone diagnostic cutoff for MCI. For candidates scoring 24–25 after education correction, enrollment will require corroborating clinical evidence of cognitive concern and preserved basic activities of daily living consistent with MCI. The education-adjustment rule, raw score, adjusted score, education years, and clinical confirmation will be recorded in the screening log.

Exclusion criteria are as follows:

- Diagnosis of Alzheimer disease dementia, other forms of dementia, delirium, or another condition that explains cognitive impairment better than MCI.
- Severe or unstable psychiatric disorder, neurological disorder, or medical illness that would interfere with participation or outcome assessment.
- Contraindications to MRI, such as implanted metallic devices or severe claustrophobia, for participants entering the imaging-biomarker sub-cohort.
- Severe sensory, speech, physical, or functional impairment preventing meaningful interaction with the robot or tablet-based training system.
- Participation in another cognitive intervention clinical trial during the study period.
- Any condition judged by investigators to pose excessive risk or compromise protocol adherence.

### Randomization and blinding

After eligibility confirmation and baseline assessment, eligible participants will be randomized to the AI-Robot group or active control group using a computer-generated block randomization sequence with a 1:1 allocation ratio. The allocation sequence will be generated by an independent researcher who is not involved in recruitment, intervention delivery, outcome assessment, or data analysis. Allocation concealment will be maintained using sequentially numbered, opaque, sealed envelopes until the participant has completed baseline assessment and has been formally enrolled.

Because of the nature of the intervention, participants and intervention facilitators cannot be blinded to group assignment. Outcome assessors, MRI analysts, laboratory staff, and statistical analysts will remain blinded to group allocation until the primary analysis has been completed. Participants will be reminded not to disclose group assignment to outcome assessors.

### Interventions

Both groups will receive three sessions per week for 24 consecutive weeks, for a maximum of 72 scheduled sessions. Each session will last approximately 45–60 min and will target multiple cognitive domains commonly affected in MCI, including episodic memory, attention, executive function, language, and orientation. Sessions will be delivered individually in a quiet room by trained facilitators who can assist with technical issues and monitor participant safety but will not provide additional cognitive coaching beyond the protocol.

Both arms are specified according to TIDieR and CONSORT guidance for non-pharmacological interventions, with equivalent reporting of provider, setting, dose, session structure, facilitator contact, permitted assistance, materials, and fidelity monitoring (31, 32). The intended contrast is the adaptive, embodied, LLM-supported tailoring component; therefore, schedule, contact time, target cognitive domains, basic facilitator presence, and visit environment will be matched as closely as feasible between groups.

### AI-Robot group: LLM-supported socially assistive adaptive cognitive training

Participants assigned to the AI-Robot group will receive cognitive training delivered by a socially assistive robot. The robot will provide spoken instructions, conversational prompts, task feedback, and session summaries. The intervention architecture comprises three layers: a robot/tablet front end for task delivery, a deterministic rule-based adaptive controller, and a version-locked Chinese-capable LLM communication module that generates supportive wording for controller-selected tailoring actions. The LLM will function as a constrained communication component within protocol-defined boundaries. It will not make diagnoses, provide medication advice, or replace clinical judgment.

Each session will follow a three-phase structure. The warm-up phase (approximately 5–10 min) will include brief social greeting, orientation questions, and low-demand cognitive activities. The core training phase (approximately 30–40 min) will include adaptive tasks targeting memory, attention, executive function, language, and orientation. The cool-down phase (approximately 5–10 min) will summarize performance, provide positive closure, and assess fatigue or distress.

Training tasks will be embedded in everyday scenarios, such as remembering appointments, following multistep instructions, semantic categorization, planning a daily schedule, and solving practical problems. Task complexity can be modified by adjusting the number of stimuli, memory load, rule complexity, interference, response-time expectations, and the amount of cueing provided.

At prespecified decision points, usually after each task block or every 5–10 min, the system will summarize task accuracy, median response latency relative to the task target, error patterns, repeated requests for help, hesitation, verbalized confusion, facilitator-rated fatigue or distress, and engagement indicators into a structured user-state vector. A deterministic rule layer will govern adaptation before any LLM-generated wording is delivered: difficulty may increase only when accuracy is at least 80% and median latency is no greater than the task-specific target for two consecutive blocks with no more than mild distress; difficulty is maintained when accuracy is 60%–79% or latency is within 100%–150% of target; and difficulty is decreased or instructions are simplified when accuracy is below 60%, latency exceeds 150% of target, two or more help requests occur in a block, or fatigue/frustration is rated moderate or higher. These thresholds are prespecified pragmatic starting rules derived from expert consensus and feasibility considerations before trial initiation; they are not treated as validated clinical cut-offs and will be documented in the intervention manual.

The adaptive logic will prioritize participant safety and sustained engagement. Affective cues will be coded on a 0–3 facilitator checklist (0 = absent, 1 = mild, 2 = moderate, 3 = severe) using observable indicators such as repeated negative verbalizations, facial or vocal signs of frustration, disengagement, and explicit fatigue. A rating of 2 will trigger de-escalation, supportive feedback, or a rest break; a rating of 3, a participant request to stop, persistent confusion despite simplification, fall risk, or facilitator concern will trigger session termination according to prespecified stop rules. The LLM can generate supportive language within these boundaries, but it cannot override rule-based de-escalation or stop decisions.

### Active control group: non-adaptive computerized cognitive training

Participants assigned to the active control group will receive non-adaptive computerized cognitive training matched to the AI-Robot group in session frequency, approximate duration, visit setting, facilitator presence, and target cognitive domains. Control tasks will cover memory, attention, executive function, language, and orientation, with the same total planned dose of up to 72 sessions. The active control will be delivered on a tablet or computer using a standardized task library and scripted instructions. Facilitators may provide technical assistance, repeat standardized instructions, and monitor safety, but they will not provide individualized cognitive coaching or motivational dialogue beyond the control manual.

Instructions and feedback in the active control condition will be standardized and limited to neutral task guidance and correctness feedback. Task difficulty will follow a predefined progression schedule based on session number and task block rather than real-time performance or emotional state. The control condition will not include user-state vector generation, JITAI-based adaptation, LLM-generated dialogue, embodied robot social behaviors, or personalized emotional support. This design is intended to compare the complete adaptive embodied intervention package with a matched non-adaptive computerized control condition while aligning session dose, cognitive domains, visit environment, and facilitator contact as closely as feasible. Accordingly, the trial evaluates the package-level effect of adaptive embodied delivery supported by bounded LLM communication, rather than the isolated effect of the LLM component or the robot interface alone.

### AI system governance, fidelity, and risk management

To align with SPIRIT-AI reporting expectations, the LLM component, prompt templates, task library, adaptive decision rules, safety filters, and system version will be locked before the first participant is randomized (14). The trial master file will record the exact model identifier, provider, release date, parameter scale when disclosed by the provider, context-window limit, deployment mode, API endpoint or local hosting configuration, latency benchmark, prompt-template version, and any external tools or retrieval resources available to the model. The model, prompt set, task library, and thresholds will be frozen during the trial unless a protocol amendment is approved. Any system change will be logged with date, rationale, version number, validation result, and potential impact on intervention fidelity.

Prompt engineering will use a template-based strategy rather than free-form prompting. Each prompt will contain only the current task context, the structured user-state vector, the permitted response categories, safety prohibitions, and a short style guide for supportive language. The rule-based controller will pass only the selected action category and permitted communication style to the LLM. The LLM output will be checked against predefined wording categories, such as simplified instruction, encouragement, rest-break wording, or facilitator-review prompts. If model output is missing, unsafe, outside the permitted wording categories, or delayed beyond the prespecified latency limit, the system will fall back to scripted non-LLM prompts and the event will be logged.

The LLM will operate within a constrained intervention architecture. Inputs will be structured summaries of task performance and behavioral cues rather than unrestricted clinical records. Outputs will be limited to supportive conversational content and formatted messages for controller-selected actions consistent with the protocol. A rule-based safety layer will block or redirect outputs outside the intervention scope, including medical advice, stigmatizing language, excessive reassurance, unsupported memory claims, diagnostic labeling, or content that could increase distress. No online learning, model-weight updating, or autonomous change to the task library will occur during the trial.

A trained human facilitator will be present during each session. Facilitators will be trained to pause or terminate the session if the participant shows distress, fatigue, confusion that cannot be resolved by simplification, or any safety concern. Fidelity will be monitored through a structured checklist covering session dose, task order, permitted assistance, adherence to adaptation thresholds, safety-layer activation, adverse events, and deviations. At least 10% of sessions per facilitator per month and all flagged interactions will be reviewed by the research team. The target fidelity threshold is at least 85% adherence to checklist items; a facilitator or system component falling below 80% in any monthly audit, or any repeated safety-relevant deviation, will trigger retraining, technical review, and corrective documentation.

Participant privacy will be protected through de-identification, access control, encrypted storage, and data minimization. If cloud-based LLM processing is used, identifiable personal information will not be transmitted unless explicitly approved by the ethics committee and covered by institutional data-processing agreements. Audio, transcript, behavioral, and system-log data will be pseudonymized before analysis, stored on access-controlled institutional servers, and handled according to the approved data management plan.

### Adherence, retention, and concomitant care

Attendance, session completion, task exposure, rest breaks, session interruptions, and reasons for missed sessions will be recorded. Participants who miss a session will be contacted to reschedule within the same week when feasible. Strategies to promote retention will include flexible scheduling, reminder calls or messages, transportation guidance where available, and regular check-ins about fatigue and burden.

Participants will continue to receive usual care during the trial. Initiation of other structured cognitive training programs or participation in another cognitive intervention trial during the study period will not be permitted. Changes in medications, medical diagnoses, or relevant clinical events will be recorded and considered in sensitivity analyses when appropriate.

### Outcome measures

The schedule of enrollment, interventions, and assessments is summarized in Table 1. The adaptive intervention components, decision rules, permitted actions, and safety and fidelity controls are summarized in Table 2. Outcome measures will be administered by assessors blinded to group allocation. Validated Chinese versions of instruments will be used where available.

**Table 1:** Schedule of enrollment, interventions, assessments, and post-intervention follow-up.

Study item	Screening/enrollment	Baseline	Weeks 1-24 intervention	Week 24 post-intervention	Week 36 follow-up
Eligibility screening	X				
Informed consent	X				
Demographics and medical history		X			
Randomization		X			
AI-Robot adaptive training			X		
Non-adaptive active control training			X		
MoCA primary outcome		X		X	X
Domain-specific cognitive battery		X		X	X
Emotional symptoms, motivation, usability, acceptance, expectancy, novelty, and social-presence measures		X	selected session-level logs	X	X
Adherence and engagement logs			continuous	summary	
Adverse-event monitoring			continuous	X	X
rs-fMRI and plasma biomarkers in nested sub-cohort		X		X	

**Table 2:** Adaptive intervention components, decision rules, safety controls, and evidence/rationale.

Component	Decision rule or variable	Permitted action	Safety and fidelity control	Evidence/rationale
Cognitive task difficulty	Accuracy, latency, error pattern, and help requests; progress only after ≥80% accuracy and latency ≤ target for two consecutive blocks; de-escalate if accuracy <60%, latency >150% of target, or ≥2 help requests	Increase, maintain, decrease, or simplify task demand	Rule-based thresholds; de-escalation overrides progression; task library version-controlled	Maintains challenge while reducing overload and repeated failure
Instruction and feedback	Hesitation, confusion, incorrect responses, repeated instruction requests, and need for cueing	Repeat, rephrase, simplify wording, increase cueing, slow pacing, or adjust feedback frequency	Templates reviewed before trial; no diagnostic, prognostic, medication, or unsupported memory claims	Supports comprehension and comparable task exposure
Affective-motivational support	Facilitator-rated fatigue, distress, frustration, disengagement, and negative affective cues on a 0-3 checklist	Encourage, normalize errors, reduce demand, offer rest, or pause the session when rating ≥2	Rating 3, request to stop, or facilitator concern triggers stop rules	Protects comfort and reduces distress-related dropout
LLM communication module	Controller-selected action category, structured state vector, task context, and protocol constraints	Generate supportive wording only within the controller-selected action category	Frozen model/prompt version; parser restricts wording; unsafe or delayed output falls back to scripted prompts	Personalizes communication without autonomous adaptation
Session pacing	Fatigue, distress, attentional decline, prolonged latency, and participant request	Slow pace, shorten block, initiate rest, reschedule activities, or terminate session	Stop rules, adverse-event reporting, facilitator training, and monthly audit	Balances intervention dose with safety and tolerability
Human oversight and fidelity audit	Session logs, transcript samples, safety flags, deviations, and facilitator checklist scores	Review ≥10% of sessions per facilitator per month plus all flagged interactions	Target fidelity ≥85%; <80% monthly adherence or repeated safety deviation triggers correction	Supports reproducibility, accountability, and SPIRIT-AI transparency

The primary outcome is change in global cognitive function from baseline to week 24, measured by MoCA score. Maintenance of MoCA change from week 24 to week 36 and between-group difference in MoCA change from baseline to week 36 will be analyzed as secondary exploratory durability outcomes.

Secondary cognitive outcomes will assess domain-specific performance. The planned battery includes the Auditory Verbal Learning Test for episodic memory, Trail Making Test Part A for attention and processing speed, Trail Making Test Part B and the Clock Drawing Test for executive and visuospatial function, Digit Span Forward and Backward for attention and working memory, semantic verbal fluency for language and semantic retrieval, and orientation items captured within the MoCA or locally validated measures.

Secondary emotional and motivational outcomes will include depressive symptoms measured by the Geriatric Depression Scale-15, anxiety symptoms measured by the Generalized Anxiety Disorder-7 scale, apathy measured by the Apathy Evaluation Scale, training motivation measured using the Intrinsic Motivation Inventory interest/enjoyment and effort/importance subscales or a locally validated equivalent, perceived engagement measured using the User Engagement Scale-Short Form plus session-level logs, technology acceptance using an older-adult digital health or robot acceptance questionnaire, usability measured by the System Usability Scale, and brief expectancy, novelty, and perceived social-presence items to help interpret potential expectancy and novelty effects. Behavioral engagement will also be captured through session attendance, completed tasks, time on task, response latency, help requests, rest breaks, and dropout.

Safety outcomes will include adverse events, serious adverse events, intervention-related distress, fatigue, falls or near falls during visits, clinically significant anxiety or frustration during sessions, and technical failures that affect participant safety or data integrity. All adverse events will be recorded, graded, and reviewed according to the approved study procedures.

### Neuroimaging and biomarker sub-cohort

A nested exploratory imaging-biomarker sub-cohort of approximately 50 participants (target 25 per group), subject to MRI eligibility, consent, and feasibility, will undergo rs-fMRI and plasma biomarker testing at baseline and week 24. MRI eligibility will be assessed separately, and contraindications to MRI will exclude only participation in the imaging component. Because this sub-cohort is not powered for confirmatory mechanistic inference, all imaging and biomarker analyses will be treated as exploratory and hypothesis-generating.

The rs-fMRI acquisition and preprocessing framework will be locked in an imaging manual before the first imaging visit. The minimum MRI protocol will include high-resolution three-dimensional T1-weighted structural imaging and resting-state echo-planar functional imaging on a 3.0-T scanner, with scanner model, sequence parameters, head-motion monitoring, eyes-open or eyes-closed instruction, and acquisition duration recorded for reproducibility. Preprocessing will include discarding initial volumes, slice-timing correction when applicable, realignment, co-registration, normalization to standard space, spatial smoothing, nuisance regression, temporal filtering, and motion scrubbing. Participants with excessive motion, defined *a priori* using framewise-displacement and gross-motion thresholds, will be excluded from affected imaging analyses. ROI analyses will focus on standard atlas-defined DMN and CEN regions, including posterior cingulate/precuneus, medial prefrontal cortex, lateral parietal cortex, dorsolateral prefrontal cortex, and posterior parietal executive-control regions. Whole-brain exploratory analyses may be conducted using appropriate correction for multiple comparisons.

Blood samples will be collected to measure biomarkers related to neuroplasticity and neurodegeneration. Planned markers include brain-derived neurotrophic factor (BDNF), phosphorylated tau 181 (p-tau181), and neurofilament light chain (NfL), which may provide complementary information about synaptic plasticity and Alzheimer disease-related pathology or neuroaxonal injury (33). Venous blood will be collected into prespecified tubes, processed within the laboratory manual time window, aliquoted to avoid repeated freeze-thaw cycles, and stored at −80°C until analysis. BDNF will be measured using a validated immunoassay kit, and p-tau181 and NfL will be measured using validated ultrasensitive immunoassay platforms when available. Batch structure, duplicate testing, calibration, lower limits of quantification, coefficient-of-variation thresholds, and procedures for out-of-range values will be specified before sample analysis.

### Sample size

The planned sample size is 126 randomized participants, with 63 participants in each group. The calculation is based on the primary endpoint, defined as change in MoCA score from baseline to week 24. The assumed between-group difference of 1.6 MoCA points and common standard deviation of 3.0 points were selected as a plausible intervention effect for trial planning, informed by prior computerized or multi-domain cognitive training trials and reviews in older adults with cognitive impairment (5, 23, 24). This assumption is used for sample-size estimation only and is not presented as a universal minimal clinically important difference. The study is designed to test whether the intervention can detect a plausible efficacy signal within a feasible single-center protocol, rather than to establish a definitive clinically important difference threshold. With a two-sided alpha level of 0.05 and 80% statistical power, 56 participants per group are required. Allowing for approximately 10% attrition, the target sample size is inflated to 63 participants per group, yielding a total sample size of 126 participants.

Sensitivity calculations using the same alpha, power, and standard deviation assumptions indicate that detectable between-group MoCA differences of 1.0, 1.2, 1.4, 1.6, 1.8, and 2.0 points would require approximately 142, 99, 73, 56, 44, and 36 participants per group before attrition, respectively; after allowing for 10% attrition, the corresponding targets are approximately 158, 110, 82, 63, 49, and 40 participants per group. The nested imaging-biomarker sub-cohort target of approximately 25 participants per group is feasibility-based and will be used for exploratory estimates of effect direction, variance, and brain-behavior associations rather than confirmatory hypothesis testing.

### Data management and confidentiality

All study data will be entered into a secure, access-controlled database using unique participant identifiers. Identifiable information will be stored separately from research data. Range checks, double-checking of critical variables, and audit trails will be used to improve data quality. Access will be restricted to approved study personnel.

Robot interaction logs, transcripts, audio-derived features, task performance data, MRI data, and biomarker data will be stored according to institutional requirements and the approved ethics protocol. Data sharing will follow participant consent, local data protection regulations, and Frontiers data availability requirements. De-identified data and analytic code may be made available upon reasonable request when ethically and legally permissible.

### Statistical analysis

Analyses will follow a prespecified statistical analysis plan finalized before database lock. The primary analysis will follow the intention-to-treat principle and include all randomized participants with available outcome data. A per-protocol sensitivity analysis will include participants who complete at least 80% of scheduled sessions (at least 58 of 72 sessions) and complete the week 24 primary outcome assessment.

Baseline demographic and clinical characteristics will be summarized using descriptive statistics. Continuous variables will be reported as mean and standard deviation or median and interquartile range, depending on distribution. Categorical variables will be reported as counts and percentages. Baseline comparability will be described rather than used as a basis for post-randomization exclusions.

The primary endpoint will be analyzed using a linear mixed-effects model with fixed effects for group, time, and group × time interaction, and with participant-level random effects to account for repeated measures. The primary contrast will be the adjusted between-group difference in MoCA change from baseline to week 24. Week 36 data will be included in durability models to estimate maintenance or decay of intervention effects after training completion. Age, sex, education level, and baseline cognitive severity will be included as covariates when prespecified. Effect estimates will be reported with 95% confidence intervals and *p* values.

Secondary continuous outcomes will be analyzed using linear mixed-effects models or generalized linear mixed models as appropriate. Binary or count outcomes such as completion status, adverse events, and help requests will be analyzed using generalized models with appropriate link functions. Missing data will be handled under the missing-at-random assumption in mixed models, with multiple-imputation sensitivity analyses considered if missingness is substantial or differs by group. The single primary endpoint will be tested at a two-sided alpha level of 0.05. Secondary outcome families will be interpreted with control of multiplicity using the Benjamini-Hochberg false-discovery-rate procedure within prespecified families, including cognitive secondary outcomes, emotional/motivational outcomes, engagement/usability outcomes, and safety outcomes; unadjusted and adjusted q values will be reported.

For rs-fMRI outcomes, ROI-to-ROI connectivity changes will be analyzed using within-group and between-group models. Whole-brain voxelwise analyses will use correction for multiple comparisons, such as false discovery rate or cluster-level family-wise-error correction. Biomarker changes will be analyzed using mixed-effects models or non-parametric alternatives if assumptions are not met, with false-discovery-rate correction across biomarker panels. Associations between changes in neurobiological measures and cognitive outcomes will be explored using Pearson or Spearman correlations and multivariable regression as appropriate, with clear labeling as exploratory.

All statistical tests will be two-tailed. Statistical significance for the primary endpoint will be set at *p* < 0.05. Secondary, week 36 exploratory durability, and imaging-biomarker analyses will be interpreted according to their prespecified exploratory or confirmatory status, with emphasis on effect sizes, standardized beta coefficients, or partial eta-squared values and 95% confidence intervals rather than *p* values alone.

### Ethics and dissemination

This study has been reviewed and approved by the Ethical Committee of the School of Nursing and Rehabilitation, Shandong University (Approval No. 2026-R-072; approved March 10, 2026). The trial has also been prospectively registered with the Chinese Clinical Trial Registry (ChiCTR2600125870). The trial will be conducted in accordance with the Declaration of Helsinki, relevant national ethical requirements, and institutional data protection policies. Written informed consent will be obtained before enrollment.

Participants will be informed of the study purpose, procedures, potential benefits and risks, confidentiality protections, voluntary nature of participation, and the right to withdraw at any time without affecting usual services. The main foreseeable risks are fatigue, frustration, transient emotional discomfort during cognitive tasks, privacy concerns related to digital interaction data, and MRI- or venipuncture-related discomfort in the exploratory imaging-biomarker sub-cohort. These risks will be mitigated through trained facilitators, real-time stop rules, privacy safeguards, and adverse-event monitoring.

Findings will be disseminated through peer-reviewed publication, conference presentation, and reports to relevant clinical and community stakeholders. Results will be reported regardless of the direction or magnitude of findings. Trial registry information will be kept current throughout the study.

## Discussion

This protocol describes an adaptive cognitive training intervention that integrates embodied social interaction, LLM-supported dialogue, and JITAI-based state tailoring for older adults with MCI. The design addresses a key limitation of many existing digital cognitive interventions: the inability to respond to momentary cognitive load, frustration, fatigue, or disengagement. By using a socially assistive robot as the interaction interface, the intervention aims to make cognitive training more socially meaningful and ecologically acceptable for older adults.

A major methodological contribution is the explicit separation of adaptive mechanisms from general cognitive stimulation. The active control condition is matched on training frequency, session duration, facilitator attention, visit environment, and cognitive domains, while withholding real-time state monitoring, rule-based tailoring, bounded LLM communication, embodied robot social behaviors, and affective-motivational tailoring. This comparison should help estimate the added value of adaptive embodied interaction beyond non-specific exposure to digital training, although expectancy, novelty, and perceived social presence may still differ between groups and will be measured to support interpretation.

The protocol also incorporates AI-specific governance elements. LLM versioning, prompt standardization, safety filters, constrained output categories, latency fallback rules, human oversight, transcript audit, and error-case review are intended to improve transparency and reproducibility. These procedures are essential because LLM-based systems can generate plausible but inaccurate or inappropriate content if not constrained and monitored. In this trial, the LLM is therefore treated as a bounded intervention component rather than an autonomous clinical decision-maker.

The nested imaging and biomarker sub-cohort may provide exploratory evidence about possible neurobiological correlates of adaptive cognitive training. Changes in DMN and CEN connectivity may help evaluate whether behavioral improvements are accompanied by altered network organization. Plasma BDNF, p-tau181, and NfL may provide complementary information about synaptic plasticity and neurodegeneration-related processes, although biomarker changes over a 24-week period should be interpreted cautiously.

Several limitations should be acknowledged. The single-center design may limit generalizability. Participants and facilitators cannot be blinded, and the AI-Robot intervention may differ from the control condition in social engagement, novelty, expectancy, and perceived human-likeness despite matching for dose and facilitator contact. The imaging-biomarker sub-cohort may be underpowered for small neurobiological effects. The LLM module introduces risks of technical failure, inappropriate output, prompt sensitivity, hidden bias in generated responses, reproducibility challenges across model versions, language- and culture-specific generalizability constraints, and model drift; these risks are mitigated through version control, locked prompts, safety constraints, latency fallback, audit procedures, and human supervision. Because the study is not yet recruiting, careful site preparation, facilitator training, registry updating, and intervention-manual locking will be important for ensuring protocol fidelity before enrollment begins.

In conclusion, this study will evaluate whether an LLM-supported socially assistive robot can deliver adaptive, affect-sensitive cognitive training for older adults with MCI. If effective, the approach may inform the development of scalable, personalized digital therapeutics that combine cognitive rehabilitation, human-robot interaction, and responsible AI governance.
